# Nanostructural Engineering of Nanoporous Anodic Alumina for Biosensing Applications

**DOI:** 10.3390/ma7075225

**Published:** 2014-07-18

**Authors:** Josep Ferré-Borrull, Josep Pallarès, Gerard Macías, Lluis F. Marsal

**Affiliations:** Nano-electronic and Photonic Systems Group, Universitat Rovira i Virgili, Avinguda Països Catalans, 26, Tarragona 43007, Spain; E-Mails: josep.pallares@urv.cat (J.P.); gerard.macias@urv.cat (G.M.)

**Keywords:** nanoporous anodic alumina, nanoengineering, pore modulation, RIfS, photonic barcodes, rugate filters, distributed Bragg reflectors, biosensing

## Abstract

Modifying the diameter of the pores in nanoporous anodic alumina opens new possibilities in the application of this material. In this work, we review the different nanoengineering methods by classifying them into two kinds: *in situ* and *ex situ*. *Ex situ* methods imply the interruption of the anodization process and the addition of intermediate steps, while *in situ* methods aim at realizing the in-depth pore modulation by continuous changes in the anodization conditions. *Ex situ* methods permit a greater versatility in the pore geometry, while *in situ* methods are simpler and adequate for repeated cycles. As an example of *ex situ* methods, we analyze the effect of changing drastically one of the anodization parameters (anodization voltage, electrolyte composition or concentration). We also introduce *in situ* methods to obtain distributed Bragg reflectors or rugate filters in nanoporous anodic alumina with cyclic anodization voltage or current. This nanopore engineering permits us to propose new applications in the field of biosensing: using the unique reflectance or photoluminescence properties of the material to obtain photonic barcodes, applying a gold-coated double-layer nanoporous alumina to design a self-referencing protein sensor or giving a proof-of-concept of the refractive index sensing capabilities of nanoporous rugate filters.

## 1. Introduction

Nanoporous anodic alumina (NAA) is a material of great interest in nanotechnology, because of its great diversity of applications, as well as its cost-effective and easily up-scalable production techniques [1,2,3]. Nanotechnological applications of this material can be found in a great variety of fields, such as energy [4,5,6,7], nanofabrication [8,9,10,11] or biotechnology [12,13]. In particular, biosensing is one of the fields in which NAA is most applied [14,15,16]. More specifically, NAA is especially adequate for optical biosensing, because of its properties of transparency in the visible range, photoluminescence [17,18] and high and tunable surface-to-volume ratio. Furthermore, its self-ordered structure when produced under the adequate anodization conditions confers it with photonic stop bands similar to those of photonic crystals [19].

The production of NAA has been a known process in industry for long, but it is the discovery of the self-ordering regime of the pores by Masuda and Fukuda [20] that supposed a breakthrough in the nanotechnological applications of this material. Since then, extensive research in the production of self-ordered pores in aluminum oxide and of their multiple applications has been carried out [2]. Self-ordered NAA is usually produced in two different experimental regimes, called mild anodization (MA) and hard anodization (HA). In mild anodization, a two-step process is applied in which the first step is carried out at a potentiostatic regime for a long time. The pores start to nucleate on the aluminum surface, and as they grow with the formation of the porous layer, they self-arrange in a triangular/hexagonal lattice [20,21]. Subsequently, this first layer is dissolved, leaving a prepatterned aluminum surface with a self-arranged array of concavities. In a second anodization step carried out at the same conditions as the first one, the pores start to nucleate at these concavities and form a self-arranged array. This process is usually accomplished with three kinds of acid electrolytes (oxalic, phosphoric [22] and sulfuric [23]), although several other kinds of acids have also been reported to permit the production of porous anodic films (*i.e.*, malonic [24], tartaric [25] or citric [26]). On the other hand, in the hard anodization regime, a higher voltage is applied to achieve a higher porous anodic layer growth rate [27]. The self-ordering of pores can be achieved without the two-step process in oxalic [28] and sulfuric [29] electrolytes. In this hard anodization regime, a danger exists because of the burning of the sample at the high voltage. For this reason, it is necessary to perform a pre-anodization step at mild anodization conditions for a short time to obtain a protective oxide layer and then increase the voltage gradually until a hard anodization voltage is reached. However, this protective oxide layer can be an inconvenience in the further application of the NAA, and an alternative method of hard anodization without the formation of the protective layer has been proposed [30] in which the anodization is performed in two steps: a first sacrificial step at hard anodization conditions to obtain adequately self-ordered concavities on the aluminum surface and then a second step at which a hard anodization voltage is applied from the very start, but at a very low temperature, low acid concentration and high stirring rate for faster heat diffusion. This way of beginning the second step promotes the correct nucleation of the pores without the need of a protective layer. Then, the acid concentration is increased gradually, until adequate hard anodization conditions are reached to complete the process.

Optical biosensing with NAA is usually carried out with the material in the form of a thin film porous layer, which can be considered a 2D nanostructured material. This strategy permits one to accurately measure the material’s effective refractive index (or the effective optical thickness of the layer) and its variation when different biological or chemical species infiltrate the pores or bind to the inner pore surface. The measurement of this effective refractive index change is done by different methods: (i) using the NAA film as a waveguide with the corresponding modes as transduction variable [31,32,33]; (ii) measuring the shift in the Fabry–Pérot oscillations in the reflectance spectrum of the NAA thin film [34]; or (iii) applying the Fourier transform to the reflectance or the photoluminescence (PL) spectrum to obtain the effective optical thickness of the layer [17,35,36].

Structuring the NAA in the third dimension can provide new properties to the material and new possible applications, or it can lead to an improvement in the existing ones. Many efforts have been reported on technology development for in-depth pore nanostructuring: Wang *et al.* applied a cyclic voltage profile in the mild anodization regime to obtain 3D-structured NAA with distributed Bragg reflector (DBR) properties [37,38,39]. Lee *et al.* applied a method to engineer the structure of NAA consisting of abruptly switching the anodization voltage from mild to hard anodization conditions in short pulses in order to avoid sample burning [28]. Losic *et al.* applied an alternative cyclic anodization current that switched continuously between mild and hard anodization conditions to obtain different kinds of serrated-like and inter-connected pores [40].

In this work, we aim at reviewing the different techniques proposed in the literature for the in-depth structural engineering of NAA and for the application of the obtained engineered nanostructures to different aspects of biosensing, in particular to optical biosensing. Furthermore, we will introduce new and different techniques developed in our group for such in-depth engineering and our proposed applications. In Section 2, we review the nanostructuring methods, classifying them as *ex situ* and *in situ*, depending on the number of steps in the production process and on the kind of fabrication parameter variations between such steps. We show a study of the influence of drastically changing one of the anodization conditions (anodization voltage, acid used in the electrolyte or acid concentration) between two different anodization steps. Then, a method to obtain distributed Bragg reflectors in a single anodization process and to tune their photonic stop bands is presented, followed by a refinement of such a method to obtain rugate filters. In Section 3, we report on the different applications of the in-depth nanoengineered NAA, and we give specific examples of using the nanostructured materials for biosensing: (i) the definition of photonic barcodes from photoluminescence or the reflectance spectra of NAA; (ii) the improvement of sensitivity in two-layer NAA structures for the detection of proteins; and (iii) the proof of concept of a refractive index sensing device using the central wavelength of a rugate filter stop band.

## 2. Nanoporous Anodic Alumina Pore Engineering

The basic pore structure of nanoporous anodic alumina consists of straight parallel pores, usually with a high aspect ratio, self-ordered in a triangular arrangement. The modification of the in-depth straight profile opens the possibility to improve the applications of this material or even to conceive many different new ones. Thus, pore engineering consists of the variation of the usual fabrication conditions (potentiostatic or galvanostatic, single- or two-step) in order to obtain the desired pore modulation. In this work, we divide the different strategies to obtain this pore engineering into two kinds: *ex situ* and *in situ*. By *ex situ*, we mean that the anodization process must be interrupted at some time and that the anodization conditions are changed drastically, namely by a change of electrolyte kind or concentration [41], a change of the kind of process (mild to hard or *vice versa* [28]) or the application of an intermediate additional non-anodizing step (like a pore widening step [42]). On the other hand, *in situ* processes are those that permit one to obtain the in-depth pore modulation by changing, without interruption and in real time, the conditions during the anodization process (current, voltage, temperature, electrolyte composition, *etc.*). The mechanisms of pore modulation are directly related to the pore formation mechanisms. It is widely accepted that the pore formation is driven by the electric field at the pore bottom and through the barrier layer [43], which is responsible for the drift of ionic species, which leads to the formation of aluminum oxide at the oxide-metal interface. At the same time, the characteristic dimensions of the porous alumina are also related to the plasticity of the barrier layer and to the need of stress relief as it is being formed [27,44,45,46]. Pore modulation arises from the change of anodization conditions with respect to the growth dynamic equilibrium. In *ex situ* methods, this change in anodization conditions is abrupt, and thus, it produces a radical change in pore distribution and morphology. On the other hand, for *in situ* methods, the change in conditions is usually gradual, and both the changes in electric field within the barrier layer, as well as the stress relief produce a continuous change in the pore diameter.

In this section, we show a systematic study of *ex situ* methods consisting of three steps in which the change of anodization parameters between the second and the third leads to the formation of structures designated as hierarchical. Next, we introduce an *in situ* method to obtain pore modulation by the cyclic variation of the anodization voltage and a further pore widening step after anodization. Finally, we introduce an alternative method for pore modulation by the cyclic variation of the anodization current, and we analyze the differences with respect to the cyclic voltage process.

### 2.1. Ex Situ Strategies: Hierarchical Nanopore Structures

Stopping the anodization process and changing drastically (although carefully and in the adequate sense) the anodization conditions leads to a great versatility in the production of NAA pore nanostructures. The first examples of *ex situ* strategies can be found in [27], where Lee and its coauthors first propose the HA technique and then propose a pore modulation strategy by alternating HA with MA with the adequate anodization conditions. At the same time, He *et al.* [47,48,49] propose an *ex situ* method based on several anodization steps intercalated with pore widening steps to achieve stepwise conical nanostructures with coded lengths that can be used as markers in biosensing. A similar approach was used by Nagaura *et al.* [50,51] to produce inverted cone structures in multistep anodization and pore widening, which they used to produce mesoporous Santa Barbara Amorphous-15 (SBA-15)-type films by sol-gel techniques. A different *ex situ* process is used to obtain multitiered three-dimensional nanostructures [52], where they produced three-dimensional structures by sequentially stepping down the anodization voltage and adequately etching the barrier layer between voltage steps. A further refinement of these procedures is to change both the voltage and electrolyte acid [53], which permits enlarging or shrinking the pores with the depth. Finally, Li and coworkers [54,55] report a very accurate control of the tapered pore profile to establish the necessary conditions of voltage variations, acid mixtures in the electrolyte and intermediate pore widening steps. More recently, *ex situ* pore 3D nanoengineering was obtained by microstructuring the aluminum previous to anodization, both by chemical etching in CuCl_2_/HCl [56,57] or by a previous lithographic step [46]. In our group, we studied how the change in a given parameter (anodization voltage, electrolyte composition or concentration) influences the pore architecture in depth, and we established rules for the realization of the desired nanostructure.

In our study, four primary anodization conditions in the potentiostatic regime are chosen (specified by the acid in the electrolyte, its concentration and the anodization voltage): (i) H_3_PO_4_ 0.3 M at 170 V and 5 °C (MA); (ii) H_2_C_2_O_4_ 0.3 M at 40 V and 5 °C (MA); (iii) H_2_C_2_O_4_ 0.3 M at 120 V and 0 °C (HA); and (iv) H_2_SO_4_ 0.3 M at 18 V and 5 °C (MA). The experiment consisted in applying one of these conditions as the first step of anodization. If this first step corresponds to an HA procedure, a protective oxide layer is previously grown on the aluminum in order to prevent the breakdown of the porous layer when the high anodization voltage is applied, as usual in HA [58]. Then, the fabricated oxide layer is dissolved by wet chemical etching in a mixture of phosphoric acid (H_3_PO_4_ 0.4 M) and dichromic acid (H_2_Cr_2_O_7_ 0.2 M) at 70 °C. In this way, a pattern of hexagonally-arranged concavities is produced on the aluminum surface, which is the starting surface for the second anodization step. This is carried out under asymmetric anodization conditions (*i.e.*, one or two anodization parameters are changed with respect to the first step). In this way, pores grow inside the concavities, and a layer of NAA is obtained. The anodization is carried out until the nanoporous anodic alumina layer is thick enough to handle. After anodizing, the remaining aluminum substrate is removed in a saturated solution of cupric chloride and hydrochloric acid (HCl/CuCl_2_), so that the bottom of the porous layer can be examined by ESEM. In order to improve the pore visibility in the SEM pictures, the pores are slightly widened by wet chemical etching in phosphoric acid solution (5 wt%) at 35 °C.

**Figure 1:**
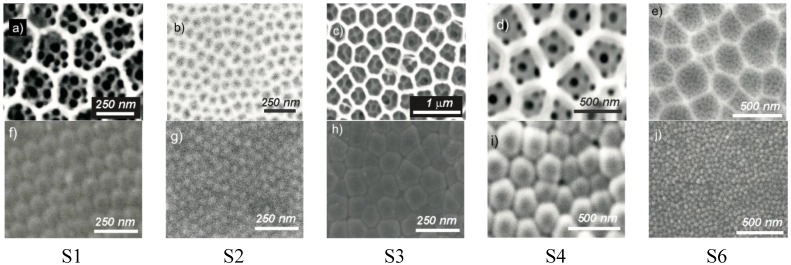
Examples of *ex situ* pore engineering: hierarchical nanopore structures. (**a**–**e**) Top SEM view of the samples indicated with the labels on the bottom; (**f**–**j**) Bottom SEM view. The fabrication conditions are specified in Appendix Table A1. Adapted with permission from [41]. Copyright 2011 WILEY-VCH Verlag GmbH & Co. KGaA, Weinheim.

**Figure 2:**
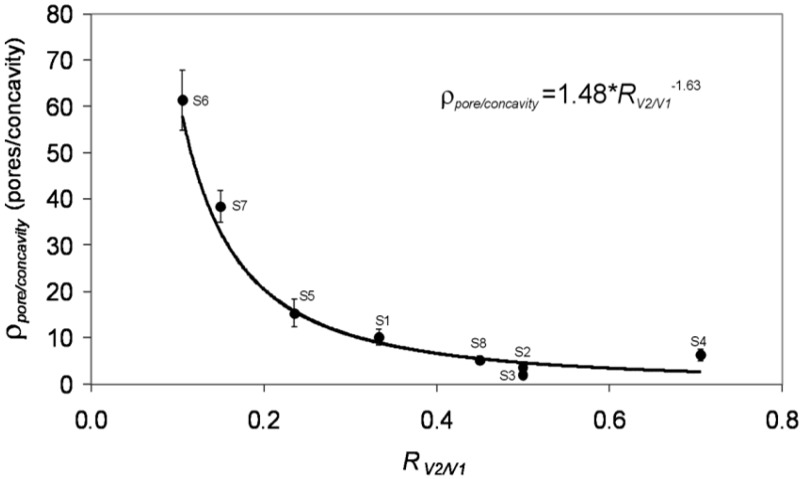
Effect of the asymmetry in the anodization voltage. The dependence of the ratio of the number of pores in the third step to the number of pores in the second step (concavities) with respect to the voltage ratio. Adapted with permission from [41]. Copyright 2011 WILEY-VCH Verlag GmbH & Co. KGaA, Weinheim.

Ten combinations of two of these four primary conditions were chosen in order to study the different influences of the change of the anodization parameters. Appendix Table A1 summarizes the details of the chosen combinations. Figure 1 shows some examples of the obtained samples for five of these combinations. The first row corresponds to the top view of the samples, while the bottom row corresponds to a bottom view of the same sample as in the first row, in order to enable the visual comparison of the geometric features of the concavities and of the pores. In all of the cases shown in this figure, it can be seen that it is possible to create pores within the concavities and that the pore density (the number of pores per concavity, designated as ρ_pore/concavity_) depends on the asymmetry in the anodization processes. Appendix Table A2 reports the estimated values of the different geometrical parameters of the obtained NAA. A remarkable observation that can be extracted from the data in Appendix Table A2 is the dependence of the parameter ρ_pore/concavity_ with the ratio between the anodization voltages in the first and the second step (V_2_/V_1_). This parameter accounts for the average number of pores produced in the second step per concavity produced in the first step and is a figure of merit for the pore density. Figure 2 shows the estimated values of this pore density as a function of the voltage ratio for the considered conditions. The data show that a clear empirical power dependence can be extracted with a power of 1.6, remarkably smaller than the power of two that could be expected if a linear relation between voltage and interpore distance were assumed.

**Figure 3:**
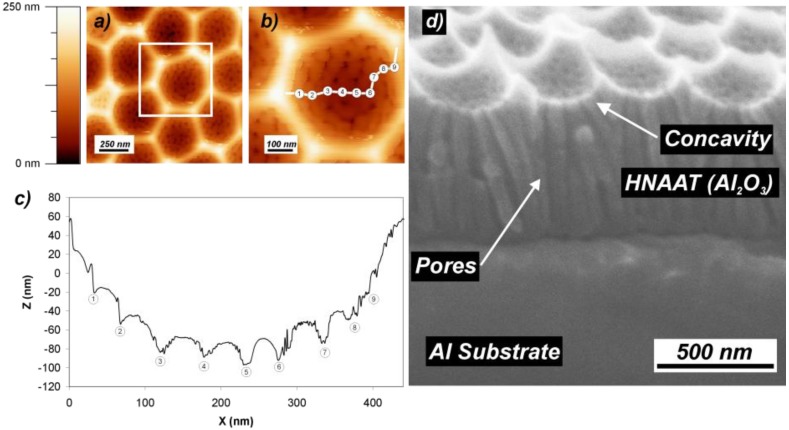
The effect of the asymmetry in the kind of acid. The new pores start to nucleate at evenly-spaced sites corresponding to the curved surface of the concavities. The pores starting nearest to the concavity rim tend to coalesce or to stop growing in order to adapt the number of pores to the flat surface. Adapted with permission from [41]. Copyright 2011 WILEY-VCH Verlag GmbH & Co. KGaA, Weinheim. (**a**) AFM top measurement of a group of concavities; (**b**) Close-up in the central concavity showing the pores; (**c**) Height profile corresponding to the path within the concavity shown in (**b**); (**d**) Cross-section SEM view of the pore structure and the nucleation at the concavities.

Figure 3 shows an example of the effect of changing the acid in the electrolyte between the first and the second steps. The figure corresponds to sample S5 obtained with a first step of phosphoric acid in MA conditions and a second step of oxalic acid in MA conditions, as well. These conditions involve both the change of acid in the electrolyte and the corresponding anodization voltage to meet the MA conditions. This leads to the creation of pores with a smaller size than the concavities. The AFM pictures in Figure 3a,b and the corresponding height profile in Figure 3c show that the new pores do not nucleate uniformly within the concavity. In the bottom of the concavity, pores grow regularly as they would in a symmetric anodization process. However, as the new pores approach the upper concavity rim, they nucleate at more spaced points, until no pores at all nucleate at the very boundary between concavities. This is in good agreement with previous observations [59] that suggest that the growth of pores is inhibited if the aluminum presents spikes or crests. Furthermore, as the concavity surface is bigger than its projection onto the surface sample, the number of pores created within the concavity exceeds the number of pores corresponding to an equilibrated growth of NAA. This makes the pores created in the upper parts of the concavity to vary their growth direction and to coalesce shortly after they are created, as shown in Figure 3d.

### 2.2. In Situ Strategies: Pore Modulation by Continuous Variation of Anodization Parameters

The variation of the anodization conditions in real time as the anodization procedure is carried out and without any interruption has the advantage of permitting one to reduce the complexity of the process of pore engineering and making it more reliable and up-scalable. Although it does not offer the many possible architectures and the versatility that *ex situ* strategies permit, it still permits a great variety of structures and applications based on a cycling of the variations in the anodization conditions, which would be extremely inconvenient for *ex situ* methods. The first reported results on in-depth nanoengineering correspond to investigations on the non-steady growth of the pores: tree-like alumina nanopores were obtained by an exponential decrease of the anodization potential [60] or serrated nanostructures in the pores appear when the aluminum foil is mounted vertically in the electrochemical cell [61,62]. More controlled modulation was introduced later by Lee and coworkers with the pulse anodization method [63], in which nanotubes of a desired length were produced by abruptly and periodically changing the anodization current density between two values. This same approach can be used to tailor the pore structure by modulating the length and delay of the pulses [44]. More complex structures, such as hyper-branched structures with tree-like branches and multiple root channels, are obtained by multi-step variations of anodic voltage and current during the second anodization, which causes the unstable and discontinuous growth of the barrier layer and results in the nucleated growth of the lateral branches from the trunk [64]. The combination of cyclic current anodization with an exponential profile and subsequent pore widening permits one to create periodically perforated pores with interconnected channels [65]. A widespread and common approach is the cyclic variation of the anodization voltage [66,67]. More elaborated schemes take into account the control of the anodization temperature in different ways: dramatically increasing the temperature from 4° to 95° de-stabilizes the planar anodization front and yields branched interconnections in the pores [68]; by cooling of the electrolyte, it is possible to enhance the pulse-anodization method [69]; or by direct cooling of the barrier layer with a heat sink setup at the basis of the aluminum foil, the alumina formation and dissolution rates can be modified and modulated pores obtained [70]. Other approaches can be reported, such as the controlled sprinkling of the electrolyte with a variable speed on the growing NAA [71]. Pore diameter modulations occur *in situ* even without a change in the anodization conditions: spontaneous current oscillations due to diffusion-controlled anodic oxidation of the aluminum have been reported in HA conditions under unstirred electrolyte [72]. Voltage control has been applied even to obtain perfectly uniform pore diameters [73], as it is known that under potentiostatic conditions, pores have a slight conical shape [74].

#### 2.2.1. *In Situ* Strategies: Pore Modulation by Anodization Voltage Variation, NAA-Based DBR

In our group, we studied the pore modulation by applying cyclic variations to the anodization voltage that result in a layered structure with pore morphologies changing from layer to layer. Applying variations in the anodization voltage can lead to different problems in the formation of the pore modulations: an excessive reduction of the anodization voltage (or at an excessive rate) can stop the pore growth process, while on the other side, an excessive increase can destroy the structure by electrical breakdown of the barrier layer. Furthermore, it is well known that anodization voltage has a major influence on the interpore distance [75] and that a change in real time is translated in the branching or the coalescence of pores. This means that the dependence of the in-depth pore morphology with the voltage variations might be complicated. In this sense, several works have exploited this dependence to obtain y-branched pores [76] and even distributed Bragg reflector structures [37] obtained with a cyclic variation of the voltage that takes advantage of the previously described pore branching process. In this work, we aim at studying in detail the dependence of the pore morphology with the voltage variations and its implications in further applications, such as biosensing. One fundamental outcome of our research is that we demonstrate that, to obtain useful optical properties fundamental for optical biosensing, it is necessary that the pore modulations are translated to effective optical refractive index changes and that this is only achieved after a pore widening post processing.

**Figure A1:**
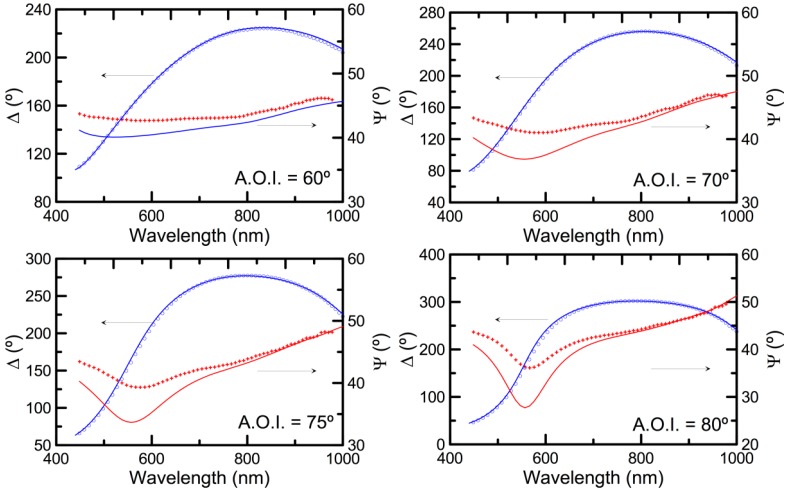
Example of the ellipsometric characterization of Sample S5 in Table A3. Ellipsometry is a technique that measures the change in the polarization state of the light upon reflection on a surface. This change is expressed in terms of two angles, Ψ and Δ (shown in symbols in the graphs). The samples are measured at four different angles of incidence (AOI = 60°, 70°, 75° and 80°) and the optical parameters (thickness and refractive index) are estimated by means of a simultaneous least-squares fitting of the four measurements with a theoretical model. The best fit is indicated with the lines in the graphs. Adapted with permission from [38].

If the pore engineering has the objective of the production of optically functional nanostructures able to translate biosensing events into measureable optical signals, it is necessary that the pore modulations generate effective refractive index differences between different layers in the nanostructure. However, porosity is weakly influenced by anodization voltage, and the pore branching obtained by its change is translated into a small variation in the effective refractive index. In order to obtain this refractive index contrast, we propose to apply post-processing to the sample consisting of a standard pore widening step for a given time length. In order to show that such a pore widening step can lead to this effective refractive index contrast, the first experiment was carried out consisting of obtaining a set of samples at different anodization voltages within the self-ordering regime of pores, in MA conditions and with the oxalic acid electrolyte. The experimental conditions are summarized in Appendix Table A3 and consist of obtaining sets of samples at voltages between 20 V and 50 V (the usual anodization potential for oxalic acid in MA conditions is 40 V) with different thicknesses. The samples were then differently etched to widen the pores (H_3_PO_4_ 5% wt at 35 °C) for different times, and the resulting layers were analyzed by spectroscopic ellipsometry. The effective refractive index and thickness of each of the samples was obtained by means of ellipsometry analysis for the different applied anodization voltages and the different applied pore widening times. Appendix Figure A1 shows an example of the ellipsometric spectra for the sample anodized at 20 V during 10 min and with no pore widening, for different angles of incidence. The figures include also the best fit on the basis of an optical model of the sample, by taking into consideration all of the angles of incidence simultaneously. With this best fit, it is possible to estimate the thickness of the sample (d = 202 nm) and its porosity (*p* = 28%). In order to validate the accuracy of the ellipsometry results, some of the NAA film thicknesses were estimated also from cross-section SEM images, and a good agreement was found between the two methods.

**Figure 4:**
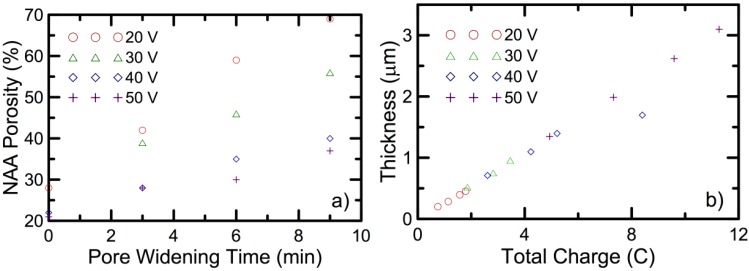
(**a**) The evolution of the porosity with the pore widening time for different anodization voltages; (**b**) The thickness of the nanoporous anodic alumina (NAA) layers as a function of the charge spent in the anodization process. Adapted with permission from [38].

The results for all of the samples show that the rate at which the porosity increases with time in the pore widening process depends on the used anodization voltage (Figure 4a): the smaller pores produced with a smaller anodization voltage show a faster porosity increase. Furthermore, this experiment permitted one also to show that the thickness of the NAA layer is linear with the amount of charge spent in the anodization process, with a proportionality constant independent of the anodization voltage (Figure 4b), which permits one to calibrate the thickness of the obtained layers from a single parameter. These results confirm that a variation of the voltage during the anodization followed by a pore widening step permit one to obtain the different layers with a refractive index contrast necessary to show complex optical properties.

**Figure 5:**
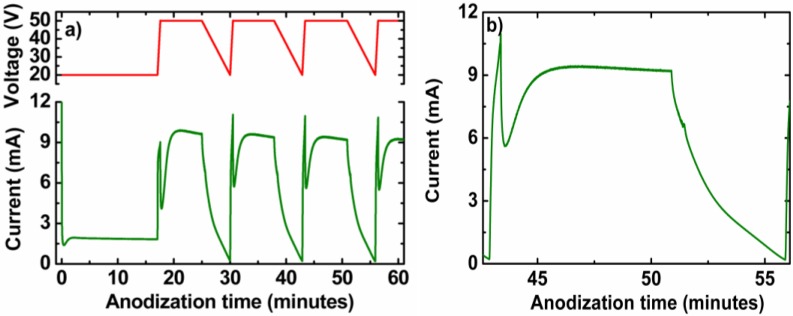
(**a**) Example of the applied anodization voltage and the corresponding measured anodization current, the first three cycles; (**b**) Detail of the third cycle. Reprinted with permission from [39]. Copyright 2013 American Chemical Society.

This results permit one to propose a nanoengineering process consisting of applying a cyclic anodization voltage profile followed by a pore widening post-processing in order to obtain NAA-based distributed Bragg reflectors. We studied the properties of the fabricated nanostructures with respect to the amount of charge spent in the process and with the pore widening time. The process to obtain these DBR samples is a standard two-step process in which the first step is performed in the usual MA conditions for oxalic acid (0.3 M and 40 V at a temperature of 6 °C) for 20 h. Then, the produced NAA is removed, and a self-arranged pattern of concavities is formed on the aluminum surface where the in-depth structured pores will nucleate. The second step consists of a voltage profile (Figure 5a, top graph) that starts with a constant voltage phase at 20 V until 2 C of charge have flowed through the system, so that the pores reach a steady growth. Then, a given number of cycles (150 in these experiments) is applied. Each cycle consists of: (i) an increasing voltage ramp with a rate of 0.5 V/s until the 50 V level is reached; (ii) a phase with a constant voltage at 50 V that lasts until a given charge Q_0_ is spent from the beginning of the phase; and, finally, (iii) a decreasing ramp with a rate of 0.1 V/s. The charge Q_0_ is used to tune the NAA thickness obtained from each cycle. The ramp rates are adjusted so that the anodization process is not stopped and that the charge used in the ramp phases is the minimum possible. The bottom graph in Figure 5a shows the current profile obtained in the anodization experiment corresponding to the voltage profile in the same figure. The data correspond to a sample with Q_0_ = 4 C. Figure 5b shows the detail of the current profile for the third voltage cycle. It is interesting to remark that in each of the phases within a cycle, the current shows two different regimes, independently of the fact that the voltage is increasing, decreasing or constant. At the beginning of a phase, the current shows a fast variation that, after some time, stabilizes. This indicates that the barrier layer, which is the element in the electrochemical circuit that regulates the current-voltage relation, has a resistance to change its state of growth. This results in a delay of the current changes with respect to the voltage changes.

**Figure 6:**
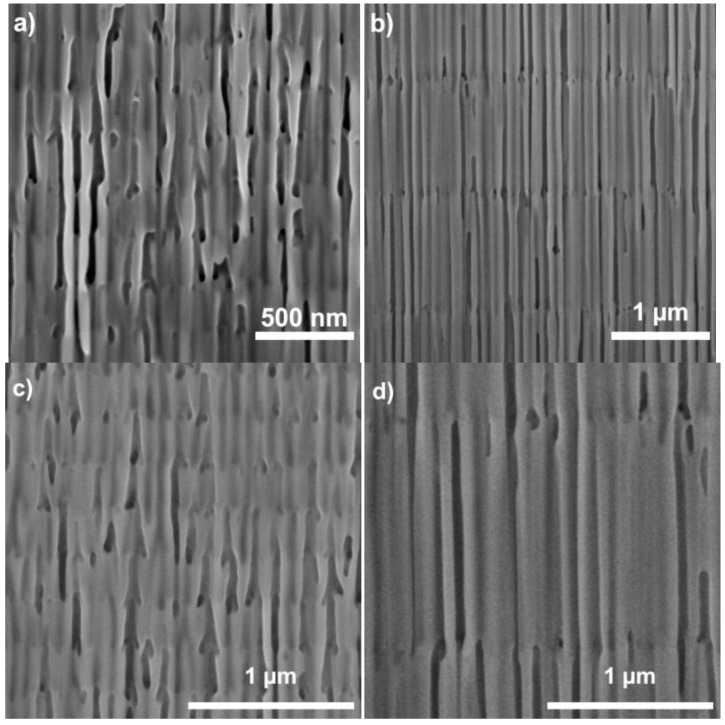
(**a**,**b**) Cross-section SEM pictures of the obtained 3D nanostructures for two different Q_0_ = 0 C and Q_0_ = 4 C; (**c**,**d**) Magnified view of the same two samples at the same scale to permit the comparison of the pore sizes. Adapted with permission from [39]. Copyright 2013 American Chemical Society.

Cross-sectional SEM pictures of two different samples (Figure 6) right after anodization and before any pore widening show that the voltage cycles are translated into change in the pore morphology in its depth. Figure 6a,c corresponds to a sample with Q_0_ = 0 C, while Figure 6b,d corresponds to Q_0_ = 4 C. Figure 6b,d is shown at the same magnification in order to permit the comparison of the pore sizes. The pores for the Q_0_ = 4 C sample show a straight profile corresponding to the constant voltage phase, while the pores for the Q_0_ = 0 C sample are clearly conical, as there is no constant voltage in any phase of the cycle. At the end of the cycle, the pores exhibit the beginning of a branching that corresponds to the lowest voltage in the cycle. This branching is frustrated in all cases, and only one of the branches survives and follows its growth, since the voltage increases immediately after reaching its minimum value of 20 V. The pictures show also that, although the interfaces between cycles can be clearly distinguished, these interfaces are not perfectly flat, but wavy and rough. It is also important to point out that no clear interfaces within a cycle can be distinguished, and the changes of the pore diameters and morphology within a cycle are continuous, in spite of the fact that the changes in voltage have discontinuities in its derivative.

**Figure 7:**
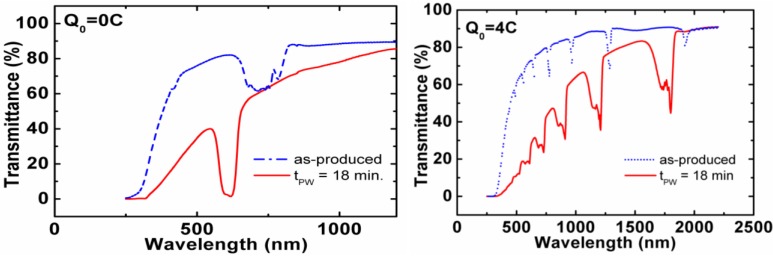
Transmittance spectra of the two samples shown in Figure 6. The spectra for as-produced samples and after 18 min of pore widening are shown. Adapted with permission from [39]. Copyright 2013 American Chemical Society.

The optical properties of the fabricated samples for Q_0_ = 0 C and Q_0_ = 4 C and their dependence with the pore widening post-processing are summarized by their transmission spectra (Figure 7). The results show that for as-produced samples, the spectra show bands with a lower transmittance, although the transmittance does not decrease as would be expected in a photonic stop band. This is explained by the fact that although the pore modulations introduce refractive index variations in the depth of the sample, this contrast is not big enough to generate the expected low transmittance stop bands. Instead, after pore widening, the stop bands become deeper as the refractive index contrast increases, because of the differential pore widening rate relation with the anodization voltage. This pore widening has the additional consequence of producing a red shift in the stop bands, because of the overall decrease of the effective refractive index. The spectra show also a general decreasing trend with decreasing wavelength in the regions not corresponding to the photonic stop bands. This can be attributed to scattering losses caused by the irregular interfaces between cycles observed in the SEM pictures.

These results show that it is possible to obtain in-depth modulated pores with enough refractive index contrast to give rise to photonic stop bands in NAA-based DBR. The central wavelength of the stop bands can be tuned by the amount of charge used in the constant voltage phase of each cycle and by the pore widening time applied after anodization. This pore widening time can also be used to modulate, up to a certain extent, the width of the stop bands.

#### 2.2.2. *In Situ* Strategies: Pore Modulation by Anodization Current Variation: NAA-Based Rugate Filters

In the previous section, we have shown the possibility to obtain NAA-based structures with tailored optical properties that can be useful in biosensing, among other applications. The results show that photonic stop bands can be obtained down to the wavelength of 500 nm and up to the near-IR. However, the voltage control method limits the minimum wavelength, because of the minimum thickness of a single cycle, defined by the increasing and decreasing ramp phases. Furthermore, the voltage control procedure has the drawback of producing rough interfaces between cycles, which lead to increased scattering losses below 400 nm that may hinder biosensing applications. It is for these reasons that we have considered the alternative method of creating pore modulations and subsequent in-depth refractive index contrast by a variation of the anodization current from galvanostatic conditions.

**Figure 8:**
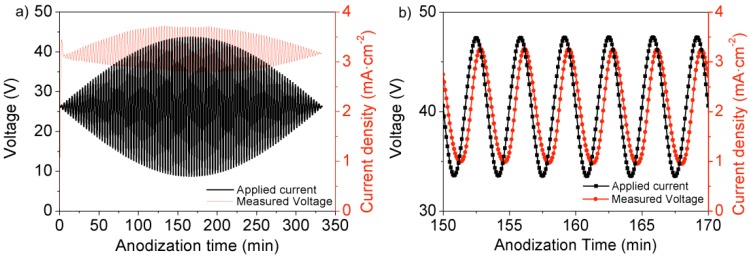
(**a**) Applied anodization current and measured anodization voltage for the fabrication of a rugate filter; (**b**) Detail of the anodization current and measured anodization voltage at the range with the highest current amplitude.

**Figure A2:**
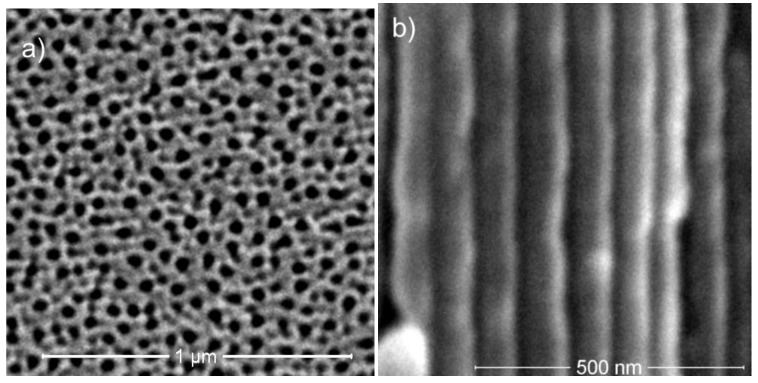
Top view (**a**) and cross-section view (**b**) of the NAA-based rugate filter structure obtained with the experimental conditions specified in the text.

More concisely, before anodization, the samples were electropolished using a mixture of perchloric acid and ethanol HClO_4_:EtOH 1:4 v/v at 20 V for 4 min. Then, the Al was anodized in H_2_C_2_O_4_ 0.3 M at 5 °C by applying a current profile with a sinusoidal variation with a period of 200 s, an average value of 2.05 mA/cm^2^ and for a total of 340 min. The amplitude of the sinusoidal variation was also modulated with the shape of the half sinusoidal period from a null amplitude at the beginning and end and up to a maximum peak-to peak value of 2.9 mA/cm^2^ at the middle of the half-period (Figure 8). This low frequency modulation aims at creating an apodized structure with reduced sidelobes in the stop bands. Finally, in order to investigate the influence of a further pore widening step in the optical properties of the nanostructure, different pore widening times in H_3_PO_4_ 5 wt% at 35 °C for 0, 5, 10 and 15 min were applied. Figure 8b shows the detail of the applied current profile and the measured corresponding voltage profile at the range with the maximum amplitude. The voltage follows also a sinusoidal profile with a delay with respect to the current changes, caused by the same barrier layer dynamics observed for the cyclic anodization voltage (Appendix Figure A2 shows a top view and a cross-section view of the obtained nanostructure).

**Figure 9:**
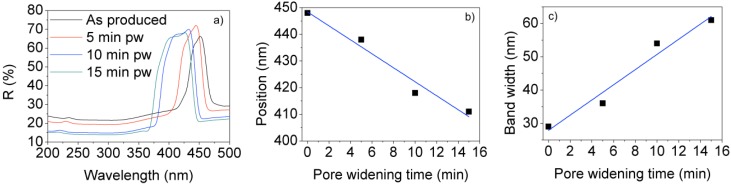
(**a**) Reflectance spectra of a rugate filter after different pore widening times; (**b**) Central wavelength of the stop band; (**c**) Width of the stop band as a function of the pore widening time.

The reflectance spectra for the as-produced samples show clear stop bands without sidelobes, as expected from the apodizing current profile (Figure 9a). It is important to note that the central wavelength for these as-produced samples is below 450 nm, which is smaller than the minimum central wavelength that can be achieved with the cyclic anodization voltage procedure. Furthermore, it can be seen that the height of the reflectance within the stop band for the different pore widening times is similar, which indicates that the as-produced structures have enough refractive index contrast to give rise to the stop bands. By examining the value of the photonic stop band central wavelength and of the stop band width in Figure 9b,c, it can be deduced that the central wavelength can be reduced and tuned with the pore widening process. On the other hand, the amplitude of the pore modulations of the consequent in-depth effective refractive index variations becomes bigger with the pore widening time, as indicated by the increase in the stop band width.

## 3. Applications of Nanoengineered NAA to Biosensing

The main wealth of literature regarding the applications of in-depth nanostructured NAA corresponds to templating applications in which NAA is used as a sacrificial scaffold to obtain nanostructures with different shapes for different applications Thus, for example, Ni nanocones can be fabricated out of *ex situ* nanoengineered NAA structures [77]. Shape coding of silica nanotubes has been demonstrated by He and coworkers [47,48,49] with an application to barcoded bioassays. *In situ* nanoengineering methods permit the fabrication of multiply-branched carbon nanotubes and nanowires [78], in particular y-junction nanotubes and nanowires, with interest in nanoelectronic devices [79,80]. The magnetic properties of ferromagnetic materials can be tailored and amplified by rendering them as modulated nanostructures [81,82]. Thermoelectric nanowires have been demonstrated by Biswas and coauthors by the use of in-depth branched NAA obtained with an *in situ* method that is selectively etched after the templating of the thermoelectric material [68]. Finally, optical properties are also modified by *in situ* nanoengineering methods, such as the DBR obtained by cyclic variations of the anodization voltage [37,66,67].

Biosensing applications of NAA are mostly based on structures with straight pores. These can be classified into three different approaches: waveguide evanescent field sensing, localized surface plasmon resonance (LSPR) sensing and reflectometry sensing. In waveguide sensing, the NAA porous structure is used to study the binding of different biomolecules. The NAA is mounted on a prism that provides the coupling of incident light, and the changes in the resonant coupling wavelength are related with the binding events in the inner pore surface [33,83]. Intensive research has been carried out on this method, leading to optimized sensing structures [31,32]. It is worth mentioning that this waveguide approach can be also used in combination with templating approaches to base the sensing on polymeric nanostructures obtained from NAA [84]. Using NAA as templates to obtain LSPR sensors is a common procedure, as the metallic nanostructures that can be obtained can be tailored in many geometrical parameters, such as length or diameter [85,86,87]. Finally, reflectometry is another method commonly used to sense changes in the molecules attached to the inner pore surface. Although one possibility is the measurement of absorbance [88], especially for gas sensing, it is the reflective interferometric spectroscopy technique that is considered the most sensitive to the changes in optical properties of the nanostructure, with examples of sensing gases [89], volatile compounds [90], tumor cells [91,92] or gold ions [93]. Nanoengineered NAA has been recently applied also to label-free biosensing applications [94,95]. Furthermore, other possibilities arise from the combination of two of these approaches: taking advantage of the photoluminescence of NAA produced under certain conditions (mainly with oxalic acid) with the waveguiding properties, a highly sensitive sensor can be obtained [96]. Covering the NAA with a small gold layer that forms caps on the oxide permits one to recognize plasmonic resonances in the reflectance spectrum that can be used to monitor different kinds of biomolecular interactions [85]. In the following, several examples of applications arising from the nanostructuring of NAA developed in our group are given.

### 3.1. Single Layer Structures: NAA-Based Barcodes

Nanoporous anodic alumina has raised great interest in biosensing, because of its optical, chemical and mechanical properties (biocompatibility, thermal stability, environmental resistance, low biodegradability), its versatility in design and its cost-effective production method. In terms of biosensing, one of the most advantageous properties is its high surface-to-volume ratio, which provides a structure with a great capability to capture analytes and, thus, to perform an amplification of the sensing signal. This fact, in combination with the material optical properties, such as its good transparency in the visible range and the photoluminescence when excited with UV light (especially for oxalic-produced NAA [97]) enables the application of even the basic NAA layers with straight pores [16,35,98,99,100]. The approach proposed in this section is based on previous work done for silicon colloids [101] or SiO_2_ microdisks [102]: NAA single layers are used to produce a unique code that can be read on the basis of its photoluminescence or its reflectance spectra and that can be used as an alternative to labeling in biosensing experiments.

**Figure 10:**
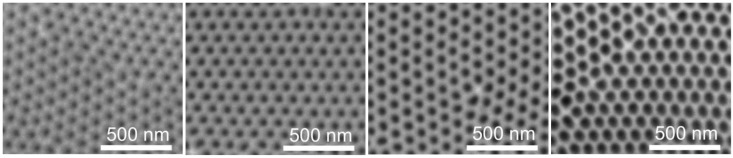
NAA with different pore radii obtained by different pore widening times. Adapted with permission from [50]. Copyright 2012 WILEY-VCH Verlag GmbH & Co. KGaA, Weinheim.

**Figure 11:**
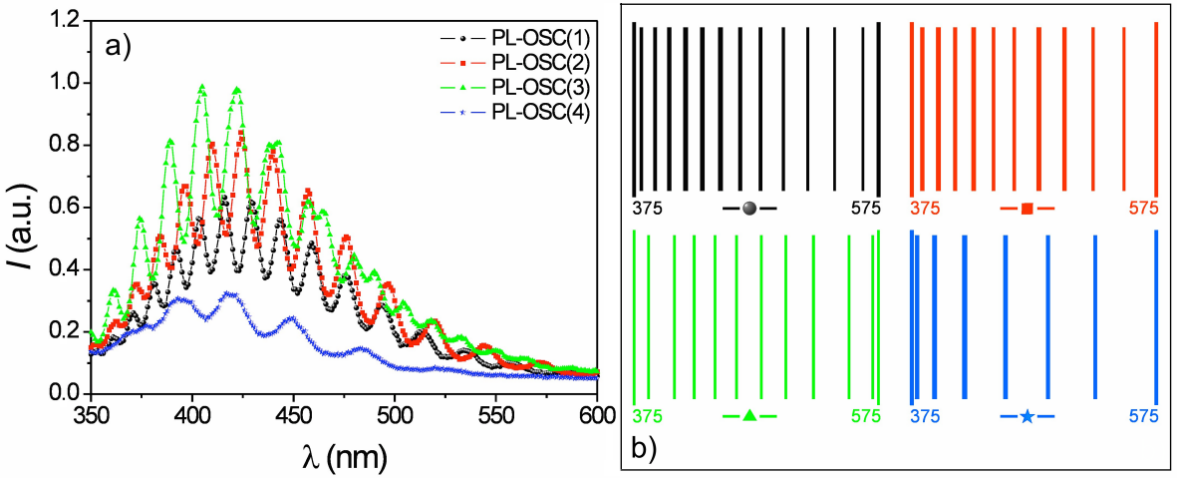
PL spectra and different corresponding barcodes. Adapted with permission from [50]. Copyright 2012 WILEY-VCH Verlag GmbH & Co. KGaA, Weinheim. (**a**) PL spectra of four samples obtained under different total charge and pore widening time conditions; (**b**) example of barcodes obtained from the PL spectra.

NAA single layers can be obtained with the desired thickness and porosity by appropriately tuning the fabrication parameters, especially the amount of charge used during the anodization and the time of the subsequent pore widening step (Figure 10). If the thickness of the resulting NAA layers is small enough to build up multiple-reflection interferences for the waves propagating at a given angle with respect to the interfaces normal, these interferences give rise to oscillating reflectance and photoluminescence spectra. These oscillations depend on the Fabry–Pérot condition:

2*n_eff_d* cosθ = *K*λ (1)

where *n_eff_* is the effective refractive index of the porous layer (which, in turn, depends on the porosity); d is the NAA layer thickness; θ is the propagation angle with respect to the interfaces normal; and λ is the wavelength; K is a natural number that establishes the condition of maximum or minimum depending on if a reflectance or a photoluminescence spectrum is considered. For instance, Figure 11a shows an example of the PL spectra of four samples obtained by the two-step MA anodization in oxalic acid. The thickness of the samples was adjusted to 5 μm, while the pore diameters were adjusted between 30 and 71 nm. The different PL spectra can be translated into unique barcodes, where the position of each bar codifies the position of each of the maxima in the PL spectrum, while the thickness of each bar codifies the PL intensity of each maximum (Figure 11b).

### 3.2. Double-Layer Gold-Coated NAA Sensors

Engineering the pore diameter in its depth provides NAA with additional features that can be advantageous for biosensing. In this section, we introduce an application of NAA bilayers (NAABs) to biosensing by means of the technique called reflective interference spectroscopy (RIfS) [103,104]. This is a refractometric technique based on the change in the reflectance spectrum from a porous thin film upon attachment of the biochemical species on the pore walls. The change in the reflectance spectrum depends mainly on the effective optical thickness (EOT) of the layer that can be evaluated from the Fourier transform of the spectrum. This effective optical thickness for a single layer is defined as EOT = 2*n_eff_d*, where *n_eff_* is the effective refractive index of the NAA layer and *d* is its thickness. The effective refractive index is the result of considering the effective medium consisting of the alumina, the medium filling the pores and also the functionalizing agents and the possible biochemical species attached to the pore walls.

**Figure 12:**
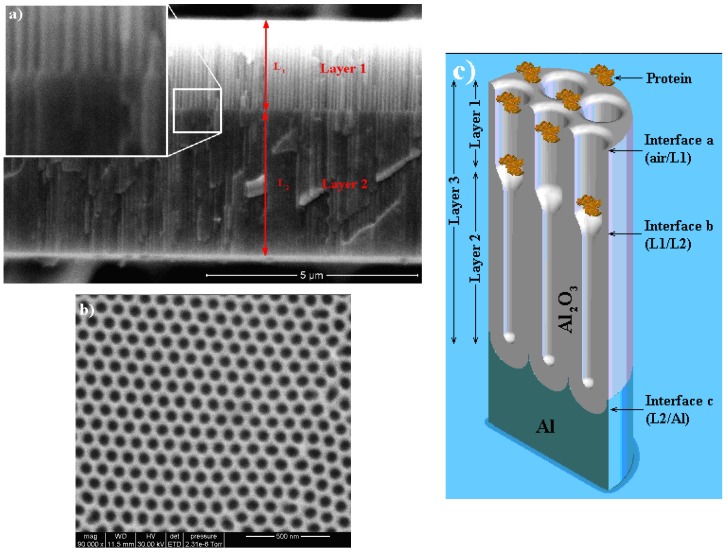
(**a**) SEM cross-section of an NAA bilayer. The inset shows a magnified view of the interface region between the two layers with different porosities to show the difference in pore diameter; (**b**) SEM top view of the sample showing the self-ordering of the pores and the uniformity of the pore distribution; (**c**) Schematic view of the sensing and self-referencing procedure, where the protein to be sensed can infiltrate Layer 1, but not Layer 2. Layer 3 corresponds to the union of Layers 1 and 2. Reprinted with permission from [55]. Copyright 2013 American Chemical Society.

A NAA bilayer is a structure consisting of pores with a variation in its diameter at a predesigned depth (Figure 12a). In order to obtain such a structure, an *ex situ* method has been applied in MA conditions in oxalic acid. Prior to anodization, an electropolishing treatment was performed in a mixture of ethanol (EtOH) and perchloric acid (HClO_4_) (4:1, v/v) at 20 V for 4 min. Next, the first anodization step was carried out in an aqueous solution of 0.3 M oxalic acid (H_2_C_2_O_4_) at 40 V and 4–6 °C for 20 h to achieve a self-ordering of the pores. The alumina layer with disordered pores was then selectively dissolved in a mixture of 6 wt% phosphoric (H_3_PO_4_) and 1.8 wt% chromic acid (H_2_CrO_4_) at 70 °C. Afterward, the second anodization step was performed under the same conditions as the first step. The anodization time during this step was adjusted to obtain the desired pore length for the high-porosity alumina layer. Subsequently, the pore diameters were widened by wet chemical etching in an aqueous solution of 5 wt% H_3_PO_4_ for 15 min. Finally, a third anodization step was carried out under the same conditions as the first step to obtain a second low-porosity alumina layer (bottom layer). Au-NAABs were obtained by depositing a 10 nm-thick gold layer on top of the NAAB through sputtering under vacuum at 30 mA for 1 min using an EMITech K575X sputter coater. With this process, the top layer has a higher porosity than the bottom layer. With an appropriate design of the pore diameters, the biochemical species (proteins in Figure 12c) can infiltrate in the top, bigger pores, but are not permitted to reach the smaller pores. With this, a self-referencing layer is created in the same substrate that is used to account for changes in the effective optical thickness that are not caused by the biochemical species to be detected, such as the influence of the solvent.

**Figure 13:**
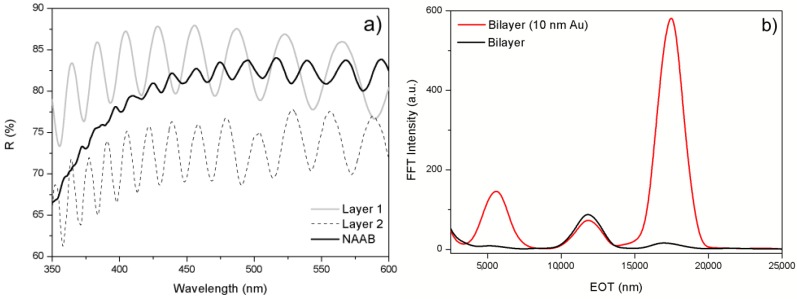
(**a**) Example of the reflectance spectra from two single layers and from the bilayer resulting from their union; (**b**) Fourier transform of NAA bilayer (NAAB) and of a gold-coated NAAB. Reprinted with permission from [55]. Copyright 2013 American Chemical Society.

However, the use of such a bilayer poses a different problem, illustrated in Figure 13a,b: in the left plot, the reflectance spectra of two single layers are shown together with the reflectance spectrum of the bilayer composed of both previous ones. The spectra for each single layer show clear Fabry–Pérot oscillations with maxima and minima that can be used to evaluate the EOT in the Fourier transform. However, the spectrum of the corresponding NAAB shows non-periodic variations with much reduced amplitude. Figure 13b (black solid curve) shows the Fourier transform of such a bilayer where two peaks can be identified: one corresponding to the bottom single layer (Layer 2 in the plot) for an EOT between 10,000 nm and 15,000 nm and a second one corresponding to the EOT of the union of the two bilayers (Layer 3 in the plot) at a range between 15,000 and 20,000 nm. A third peak is hardly visible around an EOT = 5000 nm, corresponding to the top layer (Layer 1). This reduced amplitude of the Layer 1 peak is attributed to the fact that there is a small refractive index contrast between this layer and both the bottom layer and the top incident medium. The absence of such a peak makes difficult the use of the bottom layer as the reference EOT in biosensing RIfS experiments. A solution to this problem is to increase the refractive index contrast between the top layer and the incident medium. This can be accomplished by the deposition of a very thin gold layer (10 nm) on top of the NAAB (and, thus, to obtain gold-coated NAABs, Au-NAAB). This gold layer is thick enough to increase the inner reflectivity of the NAAB and, thus, to increase the contrast of the Fabry–Pérot oscillations and to improve the visibility of the Fourier transform peak for Layers 1 and 3 (Figure 13b, red solid curve).

**Figure 14:**
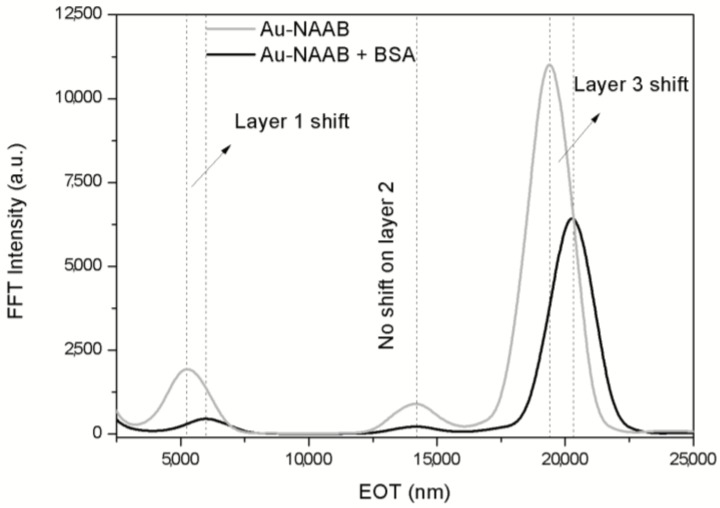
Example of the application of the Fourier transform to detect the binding of BSA inside the pores: the peaks for Layer 1 and Layer 3 show a shift in effective optical thickness (EOT), while Layer 2 (the bottom layer in the NAA bilayer) can be used as the reference. Reprinted with permission from [55]. Copyright 2013 American Chemical Society.

This improvement leads to the possibility of using the Au-NAABs for the accurate detection of bovine serum albumin (BSA) in a phosphate buffer (PBS) solution. Figure 14 shows the RIfS output for the same Au-NAAB after incubation in PBS with BSA (1 mg·mL^−1^) or in PBS alone. The results show a shift in the position of the peaks for Layer 1 and for Layer 3 for the sample incubated in the presence of BSA, while the peak for Layer 2 (the bottom low porosity layer) has no shift and can be used effectively as a self-reference signal. Furthermore, it can also be shown that the ratio of the peak intensities between Layer 1 or 3 and Layer 2 (used as the reference intensity) are even more sensitive to effective refractive index changes that the peak shifts.

### 3.3. Optical Microcavity Rugate Filters

In Section 2, we demonstrated the possibility of obtaining NAA-based rugate filters with a cyclic variation of the anodization current around an average value. This procedure permits one to achieve photonic stop bands with a central wavelength below 500 nm and with well-defined stop band limits and without sidelobes. These characteristics of the photonic stop band permit an accurate measurement of any change in the central wavelength or in the stop band width. In order to show this capability, in this section, we show an estimation of the sensitivity in the measurement of the refractive index of different liquids (water and ethanol) that infiltrate the pores completely.

**Figure 15:**
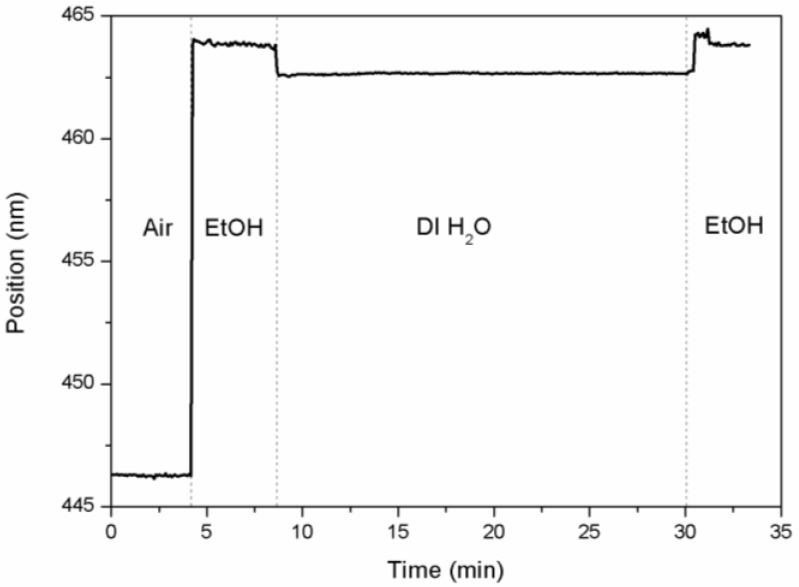
The evolution of the central wavelength of a rugate filter with time as its pores are filled sequentially with air, ethanol (EtOH) and deionized water (DI H_2_O).

The experimental setup is analogous to that of the RIfS experiments [104,105]: a CCD fiber optic spectrometer with a deuterium/halogen light source and an optical fiber reflection probe. The reflectance spectra are taken at 20-s intervals with a continuous flow of the liquids through the sample in a flow cell. The central wavelength of the stop band depends on the substance filling the pores (Figure 15): as the flow cell is filled with ethanol (EtOH, refractive index n_EtOH_ = 1.367 at λ = 465 nm), the central wavelength increases rapidly from λ_central_ = 446.3 ± 0.04 nm (average and RMS variations) up to a value of λ_central_ = 463.9 ± 0.08 nm, in accordance with the increase of the effective refractive index of the structure. Then, deionized water (DI H_2_O, *n*_H20_ = 1.336 at λ = 465 nm) is flown through the cell and replaces the ethanol, with the corresponding decrease in the central wavelength, until a stable value of λ_central_ = 462.7 ± 0.03 nm is reached. Finally, ethanol is flown again to check that the central wavelength reaches the same level as in the first infiltration.

The difference between the measured values of the central wavelength give an estimate of the sensitivity of this system to changes in the refractive index of the infiltrated liquid. The results show a difference in the central wavelength of 1.2 nm for a refractive index change of 0.031, which means a sensitivity of 39 nm/RIU with very low RMS variations in the determination of the central wavelength.

## 4. Conclusions

In this work, we presented new concepts on the in-depth structuring of the pores in nanoporous anodic alumina, and we gave examples of the application of the resulting 3D nanostructures to optical biosensing. We have divided the NAA nanoengineering methods into two kinds: *in situ* and *ex situ*. While *in situ* methods permit a single-step fabrication (with possible post-processing steps) that makes the process control and the up-scalability easier, *ex situ* strategies provide greater versatility in the pore geometry modulation and in further NAA applications.

We show the results of a study of the asymmetry in the different anodization steps in *ex situ* processes. Changing the anodization voltage leads to the ratio of the number of new pores per unit area with respect to the number of pores per unit area in the previous anodization step that follows a power of 1.6. We also show that with a cyclic anodization voltage or with a cyclic anodization current, it is possible to modulate continuously the pore geometry in its depth and to obtain materials with remarkable photonic properties, such as photonic stop bands.

The application of these nanoengineered NAA to biosensing is demonstrated in three examples. The control over the thickness and porosity of NAA single layers permits one to obtain unique photonic barcodes, which can be of use as markers in biotechnology and especially in biosensing. NAA bilayers with a thin film of gold sputtered on top constitute platforms for protein sensing with improved sensitivity and self-referencing. Finally, we show for the first time that the use of a rugate filter in RIfS set-ups permits one to achieve sensitivities as high as 39 nm/RIU with little noise.

## Appendix

**Table A1:** List of the samples produced to study the effect of the asymmetry in the fabrication conditions between the first and the second anodization steps. Adapted with permission from [41]. Copyright 2011 WILEY-VCH Verlag GmbH & Co. KGaA, Weinheim.

Changing parameter	Sample	First step				Second step			
		Acid	V (V)	C (M)	T (°C)	Acid	V (V20)	C (M)	T (°C)
Anodization Voltage	S1	H_2_C_2_O_4_	120	0.3	0	H_2_C_2_O_4_	40	0.3	5
	S2	H_2_C_2_O_4_	40	0.3	5	H_2_C_2_O_4_	20	0.3	5
	S3	H_3_PO_4_	170	0.3	5	H_3_PO_4_	85	0.3	5
Kind of Acid	S4	H_3_PO_4_	170	0.3	5	H_2_C_2_O_4_	120	0.3	0
	S5	H_3_PO_4_	170	0.3	5	H_2_C_2_O_4_	40	0.3	5
	S6	H_3_PO_4_	170	0.3	5	H_3_PO_4_	18	0.3	5
	S7	H_2_C_2_O_4_	120	0.3	5	H_2_SO_4_	18	0.3	5
	S8	H_2_C_2_O_4_	40	0.3	5	H_2_SO_4_	18	0.3	5
Concentration	S9	H_3_PO_4_	170	0.3	5	H_3_PO_4_	170	0.2	5
	S10	H_2_C_2_O_4_	120	0.3	0	H_2_C_2_O_4_	120	0.1	0

**Table A2:** Geometrical dimensions of the produced hierarchical nanopore structures for the samples in Table A1 with a change in the anodization voltage between the first and the second anodization steps. Parameter d stands for the distance between concavities (pores in the first step) or between pores (in the second step), while parameter ϕ stands for the concavity or pore diameter. Adapted with permission from [41]. Copyright 2011 WILEY-VCH Verlag GmbH & Co. KGaA, Weinheim.

Sample	d_inter-concavity_ (nm)	φ_concavity_ (nm)	d_interpore_ (nm)	φ_pore_ (nm)	ρ_pore_/ρ_concavity_ Pores per concavity
S1	249.3 ± 32.9	219.0 ± 18.7	105.0 ± 9.2	45.9 ± 10.4	10.1 ± 1.7
S2	104.0 ± 9.7	64.5 ± 7.8	74.4 ± 8.7	22.1 ± 3.6	1.9 ± 0.8
S3	393.1 ± 32.2	326.2 ± 31.5	126.2 ± 16.0	108.0 ± 23.3	3.6 ± 0.7
S4	405.6 ± 46.7	303.5 ± 37.0	282.8 ± 28.1	64.0 ± 16.4	6.3 ± 1.2
S5	413.6 ± 37.1	367.6 ± 30.0	97.6 ± 9.7	36.5 ± 7.0	15.3 ± 3.0
S6	397.0 ± 45.4	369.2 ± 53.3	48.1 ± 8.6	23.1 ± 3.7	61.4 ± 6.5
S7	292.0 ± 35.2	264.4 ± 41.0	47.5 ± 7.3	23.4 ± 4.5	38.4 ± 3.5
S8	104.4 ± 10.5	83.2 ± 11.5	55.5 ± 9.3	21.0 ± 3.0	5.2 ± 0.8

**Table A3:** List of the samples produced to investigate the effect of the anodization voltage on the pore widening rate. Adapted with permission from [38].

Sample	Anodization voltage (V)	Anodization time (min)	Total charge (C)	Temperature (ºC)	Pore widening time (min)
S1	20	10	0.8	5	0
S2		15	1.1	6	3
S3		20	1.6	6	6
S4		25	1.8	5	9
S5	30	10	1.2	6	0
S6		15	1.9	6	3
S7		20	2.8	7	6
S8		25	3.5	5	9
S9	40	10	2.6	5	0
S10		15	4.2	5	3
S11		20	5.2	6	6
S12		25	8.4	5	9
S13	50	10	4.9	5	0
S14		15	7.3	5	3
S15		20	9.6	5	6
S16		25	11.2	5	9
